# Use of patient‐derived tumor organoid platform to predict the benefit of postoperative adjuvant chemotherapy for poor responders to neoadjuvant chemoradiotherapy in locally advanced rectal cancer

**DOI:** 10.1002/btm2.10586

**Published:** 2023-08-16

**Authors:** Weisong Xue, Ting Wang, Jiaxin Yao, Wei Wu, Dexin Chen, Botao Yan, Xiaoyu Dong, Yuting Tang, Yunli Zeng, Yueyu He, Peihua Cao, Fangyuan Shao, Wenhua Huang, Chuxia Deng, Jun Yan

**Affiliations:** ^1^ Department of General Surgery, Guangdong Provincial Key Laboratory of Precision Medicine for Gastrointestinal Cancer Nanfang Hospital, The First School of Clinical Medicine, Southern Medical University Guangzhou Guangdong People's Republic of China; ^2^ Department of Gastrointestinal Surgery Shenzhen People's Hospital, The Second Clinical Medical College, Jinan University Shenzhen Guangdong China; ^3^ Department of Gastrointestinal Surgery Shenzhen People's Hospital, The First Affiliated Hospital, Southern University of Science and Technology Shenzhen Guangdong China; ^4^ Clinical Research Center, Zhujiang Hospital, Department of Biostatistics School of Public Health, Southern Medical University Guangzhou Guangdong People's Republic of China; ^5^ Cancer Center, Faculty of Health Sciences University of Macau Macau People's Republic of China; ^6^ Guangdong Engineering Research Center for Translation of Medical 3D Printing Application, Guangdong Provincial Key Laboratory of Digital Medicine and Biomechanics, National Key Discipline of Human Anatomy School of Basic Medical Sciences, Southern Medical University Guangzhou Guangdong People's Republic of China

**Keywords:** adjuvant chemotherapy, neoadjuvant chemoradiotherapy, nomogram, organoid, rectal cancer

## Abstract

Postoperative adjuvant chemotherapy (AC) for poor responders to neoadjuvant chemoradiotherapy (nCRT) remains debatable among patients with locally advanced rectal cancer (LARC), necessitating biomarkers to accurately predict the benefits of AC. This study aimed to develop a patient‐derived tumor organoid (PDTO) platform to predict the benefit of AC in LARC patients showing poor nCRT response. PDTOs were established using irradiated rectal cancer specimens with poor nCRT responses, and their sensitivity to chemotherapy regimens was tested. The half‐maximal inhibitory concentration (IC50) value for the PDTO drug test was defined based on the clinical outcomes, and the accuracy of the PDTO prognostic predictions was calculated. Predictive models were developed and validated using the PDTO drug test results. Between October 2018 and December 2021, 86 PDTOs were successfully constructed from 138 specimens (success rate 62.3%). The optimal IC50 cut‐off value for the organoid drug test was 39.31 μmol/L, with a sensitivity of 84.75%, a specificity of 85.19%, and an accuracy of 84.88%. Multivariate Cox regression analysis revealed that the PDTO drug test was an independent predictor of prognosis. A nomogram based on the PDTO drug test was developed, showing good prognostic ability in predicting the 2‐year and 3‐year disease‐free survivals (AUC of 0.826 [95% CI, 0.721–0.931] and 0.902 [95% CI, 0.823–0.982], respectively) and overall survivals (AUC of 0.859 [95% CI, 0.745–0.973] and 0.885 [95% CI, 0.792–0.978], respectively). The PDTO drug test can predict the benefit of postoperative AC in poor responders with LARC to nCRT.

**Abbreviations**
AC: adjuvant chemotherapy
AJCC: American Joint Committee on Cancer
ASA: American Society of Anesthesiology
AUC: area under the receiver operating characteristic curve
CEA: carcinoembryonic antigen
CI: confidence interval
C‐index: concordance index
DFS: disease‐free survival
H&E: hematoxylin and eosin
HR: hazard ratio
IC50: half‐maximal inhibitory concentration
LARC: locally advanced rectal cancer
mrEMVI: MRI‐detected extramural vascular invasion
MRI: magnetic resonance imaging
mrIMF: MRI‐detected involved mesorectal fascia
NCCN: National Comprehensive Cancer Network
nCRT: neoadjuvant chemoradiotherapy
OS: overall survival
PDTO: patient‐derived tumor organoids
PDTXs: patient‐derived tumor xenografts
RBCs: red blood cells
ROC: receiver operating characteristic
STR: short tandem repeat
TRG: tumor regression grade
ypN: pathological N stage
ypT: pathological T stage

**Translational Impact Statement**

The efficacy of adjuvant chemotherapy (AC) for locally advanced rectal cancer (LARC) after neoadjuvant chemoradiotherapy and surgery remains debate. In this study, we used irradiated rectal cancer specimens to establish organoids and conducted organoid drug test to validate their ability to predict the benefit of AC. The results showed organoid drug test accurately predicts chemotherapy response, those with organoid sensitivity are likely to benefit from adjuvant chemotherapy. Organoid drug test holds promise as an important component of precision treatment for LARC.

## 1 INTRODUCTION

Neoadjuvant chemoradiotherapy (nCRT) followed by oncological resection is the standard treatment for locally advanced rectal cancer (LARC), providing excellent local disease control^1^ and a high overall survival (OS) rate in patients showing good response.^2^ However, approximately 50% of patients respond poorly to nCRT^3^ with reduced OS rates,^2^ necessitating optimized treatment strategies. Postoperative adjuvant chemotherapy (AC) is the most common recommendation for poor responses to nCRT.^4^ However, the prognostic benefit of AC remains debatable, with variations in the choice of AC drugs across different countries and institutions.^5^, ^6^ Additionally, heterogeneous responses to AC have been observed even among patients with the same cancer stage and similar treatment regimens. Administering ineffective treatment to a subpopulation of patients who are resistant to chemotherapy not only fails to prolong survival but also increases the risk of treatment toxicity. Predicting individual responses to AC can aid oncologists in identifying patients whose tumors are sensitive to chemotherapy before treatment and inform treatment decisions after nCRT and curative surgery.^7^ However, no effective preclinical cancer models are available for identifying LARC patients with a poor response to nCRT who may benefit from AC.

The commonly used preclinical cancer models, patient‐derived tumor xenografts (PDTXs) and cancer cell lines, have major limitations in predicting chemotherapy response, as they do not reliably represent the original tumors.^8^ In addition, the cellular characteristics, including cell density and extracellular matrix composition, within PDTX models impact the properties of drug diffusion,^9^, ^10^ thereby influencing the predictive accuracy of drug response. However, emerging organoid technologies have recently opened new avenues for developing human cancer models.^11^, ^12^ Patient‐derived tumor organoids (PDTOs) are innovative preclinical models that closely mimic native tissues in vivo. They are formed through the self‐assembly of stem cells within a three‐dimensional environment, resulting in a structure that closely resembles the original tissue from which it was derived.^8^ In contrast to cell lines or PDTXs, PDTOs are established with less tissue.^13^ Moreover, they can be propagated more rapidly while retaining the original tumor's histopathological, genetic, and molecular characteristics, indicating their great potential for personalized precision medicine.^14^ Some studies have demonstrated that PDTOs can accurately predict chemotherapy and radiation responses in patients with different cancers.^15^, ^16^, ^17^ However, to date, there have been no reports on the construction of organoids using irradiated tumor specimens. Furthermore, the predictive ability of PDTOs for LARC patients with poor response to nCRT who may benefit from AC is unknown.

Therefore, we established a platform to construct organoids from residual tumors extracted from surgical specimens after nCRT. Our investigation aimed to assess the prognostic value of the PDTO drug sensitivity test and predict disease‐free survival (DFS) and OS for each patient using this platform.

## 2 RESULTS

### 2.1 PDTO derivation and characterization

**FIGURE 1:**
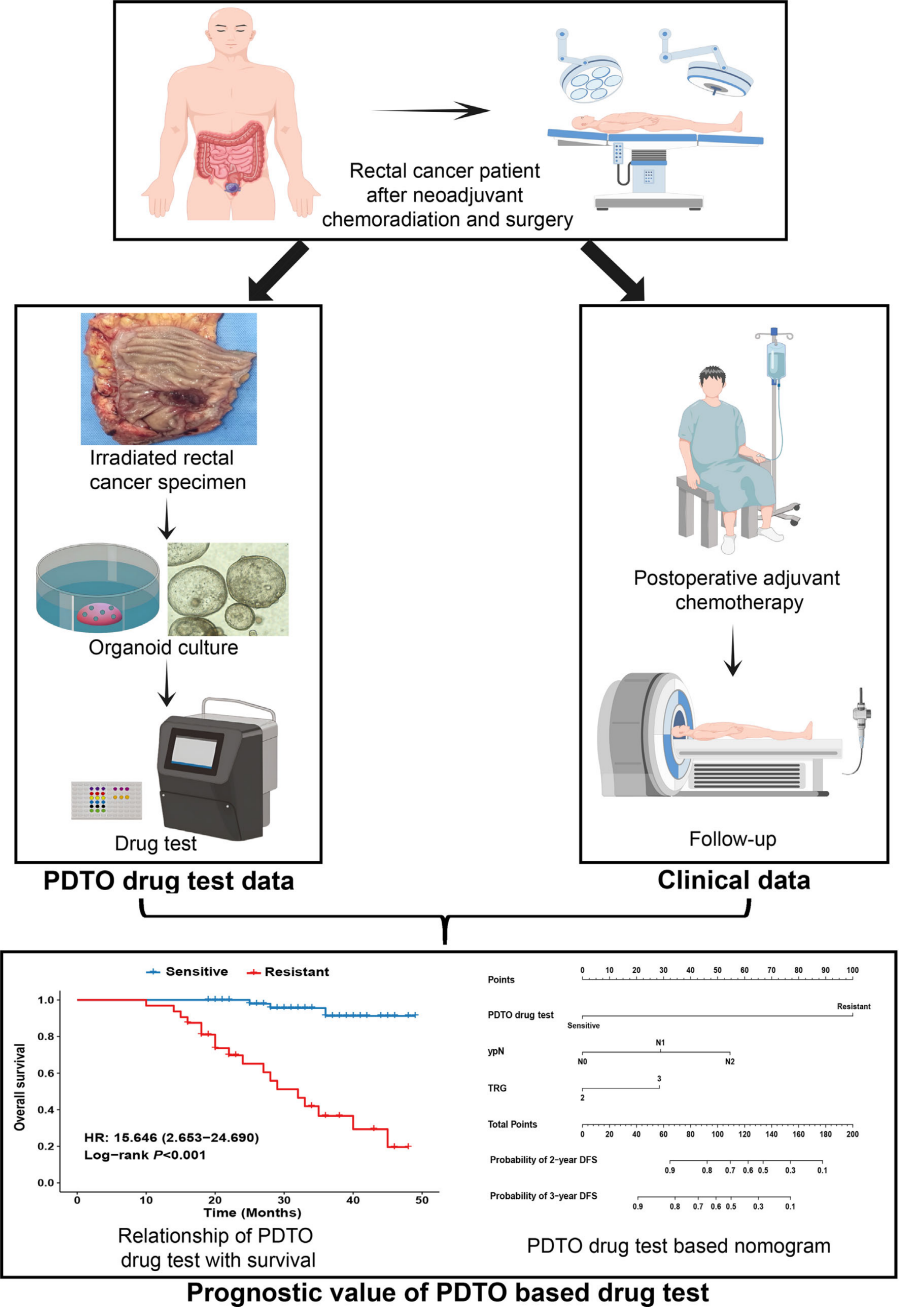
Study design. Flow diagram of the study design, including the establishment of clinical and PDTO drug test databases. PDTO, patient‐derived tumor organoids.

**FIGURE 2:**
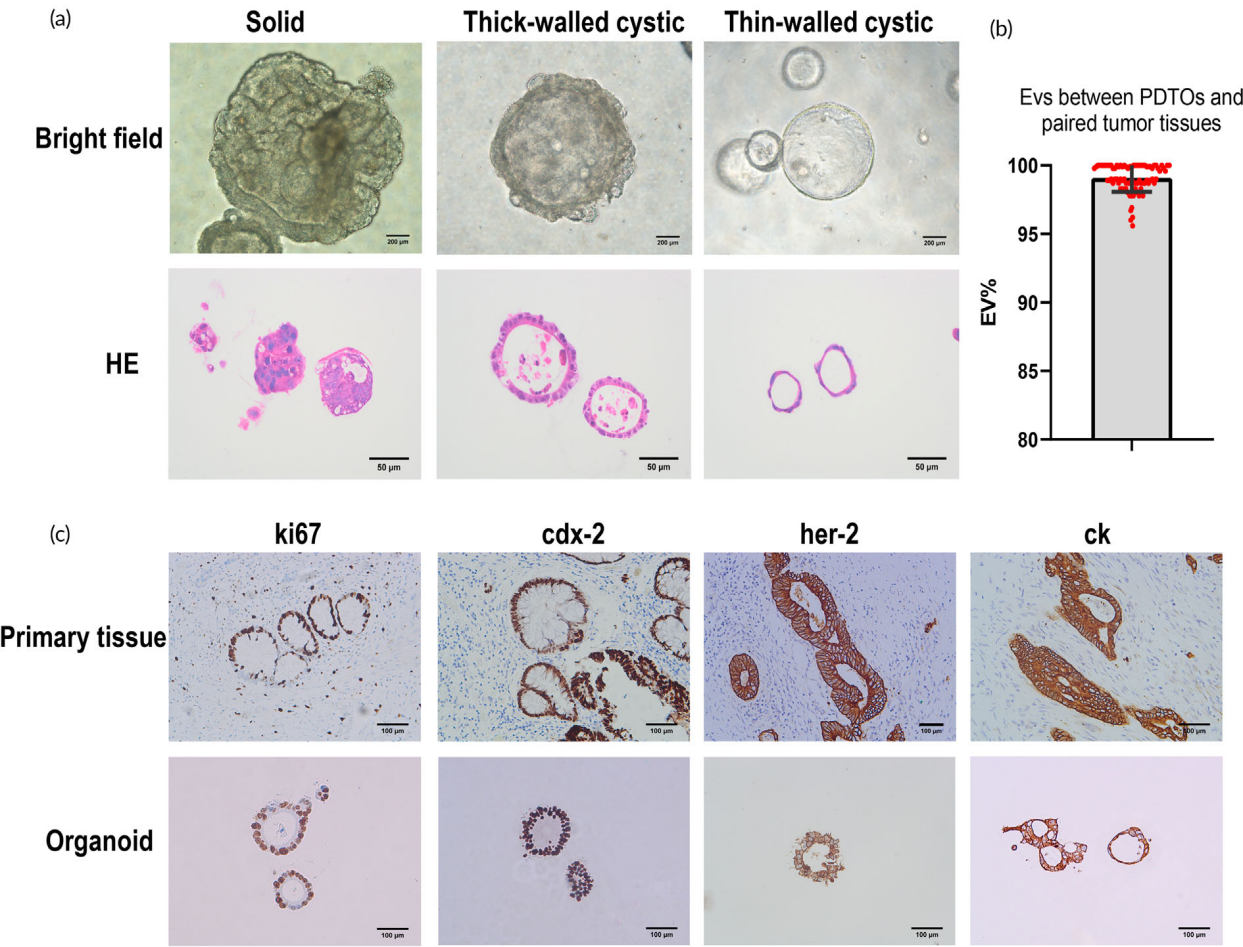
Characterization and identification of PDTOs. (a) Bright‐field images with corresponding H&E staining showing three phenotypes of PDTOs. (Bright‐field images: ×400 magnification, 200 μm scale bars; H&E staining: ×200 magnification, 50 μm scale bars). (b) Evaluation values (EVs) between the tested PDTOs and paired tumor tissues were expressed as mean ± standard deviation. EV = (number of generated peaks of a cell line from PDTOs) × 2/(total number of peaks of cell lines from PDTOs and paired tumor tissues). (c) Immunohistochemistry staining of ki‐67, cdx‐2, her‐2, and ck on PDTOs and corresponding primary tumors (×200 magnification, 100 μm scale bars). H&E, hematoxylin and eosin; PDTO, patient‐derived tumor organoids.

The study flow diagram is shown in Figure 1. Rectal cancer specimens surgically resected after nCRT at Nanfang Hospital were collected for organoid culture. Then, PDTO drug sensitivity tests were conducted. Between October 2018 and December 2021, we successfully constructed 86 PDTOs (62.3%) from 138 irradiated specimens. Of the 138 specimens, 37 (26.8%) failed to establish organoids, and 6 (4.3%) were contaminated with bacteria or fungi. Additionally, organoids were successfully cultured in nine cases (6.5%) but did not yield sufficient cells for the drug test. When observed under the bright field of the microscope, the organoid showed three main morphologies: solid, thick‐walled cystic, and thin‐walled cystic structures (Figure 2a). Hematoxylin and eosin (H&E) staining showed that tumor cells were cluster‐aggregated in solid organoids. Moreover, the tumor cells showed a columnar form on the cyst wall in thick‐walled cystic organoids and a flattened form in thin‐walled cystic organoids. Additionally, H&E staining showed that PDTO maintained tumor characteristics with an enlarged nucleus and increased nuclear atypia (Figure 2a). The median (range) of MycoAlert ratios of all examined PDTO cultures was 0.30 (0.24–0.41) (Figure S1), implying that our PDTO cultures were mycoplasma‐free.

### 2.2 Genomic and molecular characteristics are preserved in PDTOs

Short tandem repeat (STR) profiles showed 95.28% ± 3.49% concordance between the organoids and their corresponding original cancer tissue (Figure 2b), indicating high matching levels.^18^ The detailed STR test results are shown in Figure S2. Furthermore, in the marker expression analysis commonly used to diagnose rectal cancer, we observed that organoids and primary tumors showed similar expression profiles for ki67, cdx‐2, p53, pms2, her‐2, ck20, and ck (Figure 2c, Figure S3). These data indicated that the parental tumor's genomic and molecular characteristics were maintained in PDTOs.

### 2.3 Heterogeneous responses of PDTOs to chemotherapy regimens

**FIGURE 3:**
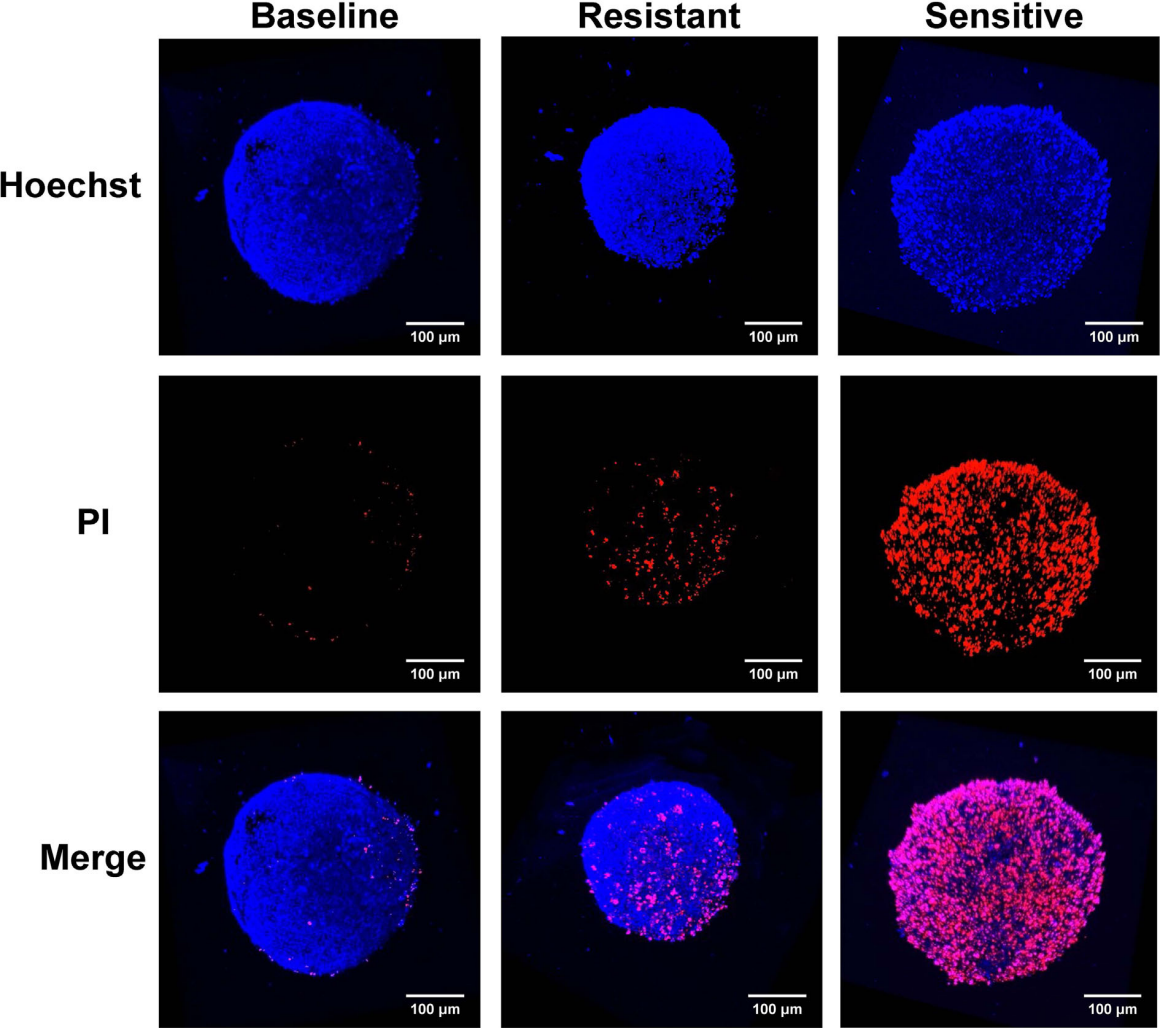
Organoid viability was assessed by immunofluorescence staining using a LIVE/DEAD cell staining kit. Confocal images of the live and dead cells stained with Hoechst 33258 (blue) and propidium iodide (red), respectively (×200 magnification, 100 μm scale bars).

We observed a heterogeneous response of PDTOs to the same concentration of chemotherapy regimen. Confocal fluorescence microscopy revealed that certain organoids exhibited higher levels of cell death at a drug concentration of 50 μmol/L, and others showed only minimal cell death (Figure 3). The number of dead cells in the drug‐sensitive organoids was significantly higher than that in the drug‐resistant organoids (Figure S4). Heterogeneous responses of PDTOs to different drug concentrations were also observed. In organoids sensitive to chemotherapy drugs, the size of organoids was significantly decreased as the drug concentration was increased, whereas the size of drug‐resistant organoids decreased slowly with increasing drug concentration (Figure 5A,B).

### 2.4 Association of organoid drug response with patient clinical response

**FIGURE 4:**
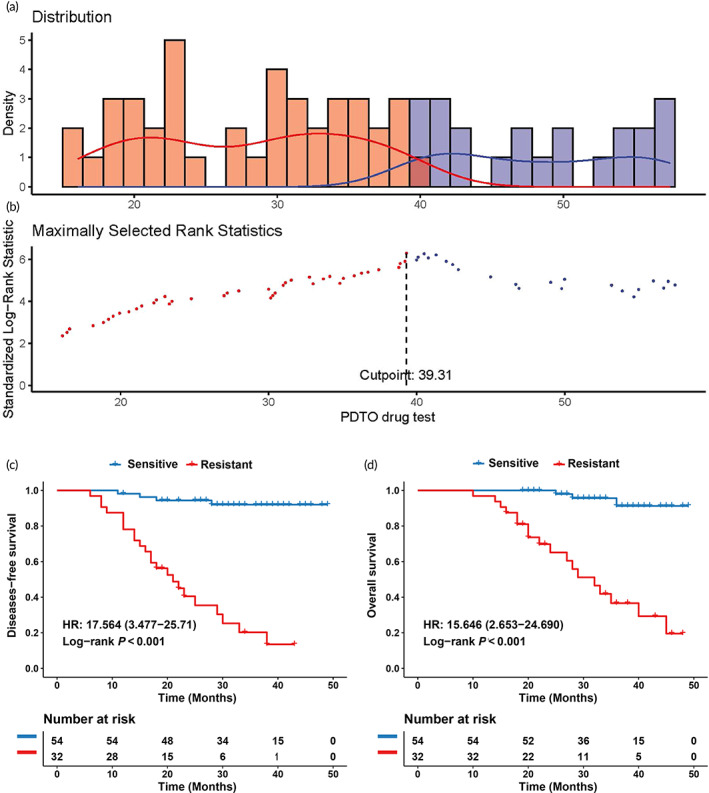
Determination of the optimal cut‐off value for the PDTO drug test and its relationship with survival. (a) Density distribution histogram for organoid sensitivity and resistance groups separated by the optimal cut‐off value. (b) Scatter plot showing standardized log‐rank statistic values for different PDTO drug test cut‐off values. *N* = 54 and 32 for patients with organoid sensitivity and resistance, respectively. The cut‐off value was determined using two‐sided maximally selected rank statistics. **(**c) The DFS rate difference between the organoid‐sensitive and resistant patients determined by the PDTO drug test cut‐off value. (d) The OS rate difference between the organoid‐sensitive and resistant patients determined by the PDTO drug test cut‐off value. DFS, disease‐free survival; HR, hazard ratio; OS, overall survival; PDTO, patient‐derived tumor organoid.

The demographic characteristics of 86 LARC patients were extracted from the database and are summarized in Table 1. Power analysis showed that this sample size achieved sufficient power for the current study (Data S1). The best cut‐off value for the half‐maximal inhibitory concentration (IC50) calculated using the “survival” package was 39.31 μmol/L, which provided the highest standardized log‐rank statistic (Figure 4a,b). Organoid resistance and sensitivity are indicated by an IC50 value ≥39.31 μmol/L and < 39.31 μmol/L, respectively. Accordingly, 54 organoids were classified as sensitive and 32 as resistant. Dose–response curves in different groups were generated based on the activity values of organoids exposed to six different drug concentrations (Figure S6). Comparing organoid drug test data with clinical data, 23 out of 32 patients in the organoid‐resistant group had tumor recurrence or metastasis, while only 4 out of 54 patients in the organoid‐sensitive group had tumor recurrence or metastasis. In addition, 18 out of 32 patients in the organoid‐resistant group died, while only 3 out of 54 patients in the organoid‐sensitive group died. As expected, the DFS and OS rates were significantly decreased in the resistant group compared to the sensitive group, with hazard ratios (HRs) of 17.564 (3.477–25.71; *p* < 0.001) and 15.646 (2.653–24.690; *p* < 0.001), respectively (Figure 4c,d). Furthermore, the defined cut‐off value for predicting the benefit of AC for poor responders after nCRT in LARC had a sensitivity of 84.75% (95% confidence interval [CI], 73.48%–91.76%), a specificity of 85.19% (95% CI, 67.52%–94.08%), and an accuracy of 84.88% (95% CI: 75.84%–90.95%), with a positive (PPV) and negative predictive value (NPV) of 92.59% (95% CI: 82.45%–97.08%) and 71.88% (95% CI: 54.63%–84.44%), respectively (Table 2). These results suggest that the defined cut‐off value of IC50 can be used to distinguish between sensitive and resistant patients effectively.

**TABLE 1:** Patient demographics and tumor characteristics.

Variable	*N* = 86
Sex, No. (%)	
Female	22 (25.6)
Male	64 (74.4)
Age, median (IQR), years	59 (54.6–65.3)
BMI, mean ± SD, kg/m^2^	22.3 ± 2.9
ASA score, No. (%)	
I	58 (67.4)
II	28 (32.6)
CEA, No. (%)	
<5 ng/mL	71 (82.6)
≥5 ng/mL	15 (17.4)
Tumor location^a^, median (IQR), mm	55.0 (40.0–66.0)
Tumor length, median (IQR), mm	25.0 (20.0–33.5)
Tumor thickness, median (IQR), mm	15.0 (10.0–20.0)
mrEMVI, No. (%)	
Yes	20 (23.3)
No	66 (76.7)
mrIMF, No. (%)	
Positive	17 (19.8)
Negative	69 (80.2)
AJCC stage before nCRT, No. (%)	
II	9 (10.5)
III	77 (89.5)
Clinical T stage, No. (%)	
cT3	38 (44.2)
cT4a	42 (48.8)
cT4b	6 (7.0)
Clinical N stage, No. (%)	
cN0	3 (3.5)
cN1	19 (22.1)
cN2	53 (61.6)
cNx	11 (12.8)
Surgery, No. (%)	
SPS	76 (88.4)
APR	10 (11.6)
Differentiation^b^, No. (%)	
Well (G 1)	15 (17.4)
Moderate (G 2)	58 (67.4)
Low (G 3)	13 (15.1)
Circumferential margin	
Positive	4 (4.7)
Negative	82 (95.3)
Vascular invasion	
Yes	16 (18.6)
No	70 (81.4)
Nerve invasion	
Yes	29 (33.7)
No	57 (66.3)
AJCC stage^c^, No. (%)	
I	8 (9.3)
II	35 (40.7)
III	43 (50.0)
AJCC TRG^c^, No. (%)	
2	50 (58.1)
3	36 (41.9)
Pathological T stage, No. (%)	
ypT1	0 (0)
ypT2	18 (21.0)
ypT3	42 (48.8)
ypT4	26 (30.2)
Pathological N stage, No. (%)	
ypN0	43 (50)
ypN1	28 (32.6)
ypN2	15 (17.4)
Organoid IC50_,_ median (IQR)	34.9 (23.0–47.5)

Abbreviations: AJCC, American Joint Committee on Cancer; APR, abdominoperineal resection; ASA, American Society of Anesthesiologists; BMI, body mass index; CEA, carcinoembryonic antigen; G, tumor grade; IQR, interquartile range; mrEMVI, MRI‐detected extramural vascular invasion; mrIMF, MRI‐detected involved mesorectal fascia; nCRT, neoadjuvant chemoradiotherapy; No., Number; SD, standard deviation; SPS, sphincter preservation surgery; TRG, tumor regression grading.

^a^ Distance of the tumor from the anal verge.

^b^ According to the (2010) WHO classification of tumors of the digestive system.

^c^ According to the AJCC on Cancer guidelines.

**TABLE 2:** Predictive ability of defined IC50 cutoff value in PDTO drug test for adjuvant chemotherapy response prediction.

*N* = 86		Response in the clinic		
		Response *N* = 59	Nonresponse *N* = 27	
Drug response in PDTO	Sensitive, *N* = 54	50	4	PPV = 92.59% (50/54)
	Resistant, *N* = 32	9	23	NPV = 71.88% (23/32)
		Sens = 84.75% (50/59)	Spec = 85.19% (23/27)	Accuracy = 84.88% (73/86)

Abbreviations: NPV, negative predictive value; PDTO, patient‐derived tumor organoid; PPV, positive predictive value; Sens, sensitivity; Spec, specificity.

### 2.5 Prognostic value of PDTO drug test

Univariate Cox regression analyses showed significant associations between DFS and magnetic resonance imaging (MRI)‐detected extramural vascular invasion (mrEMVI), MRI‐detected involved mesorectal fascia (mrIMF), American Joint Committee on Cancer (AJCC) stage, AJCC tumor regression grade (TRG), pathological T stage (ypT), pathological N stage (ypN), and the PDTO drug test (Table S1). Moreover, mrEMVI, mrIMF, AJCC stage, AJCC TRG, ypN, and PDTO drug tests were significantly related to OS (Table S1). Given the high correlation between ypN and the AJCC stage (Kendall r = 0.651, *p* < 0.001) and that the HR of ypN was higher than that of the AJCC stage, ypN was then subjected to multivariate Cox regression analysis. This analysis revealed that AJCC TRG (HR, 2.558; 95% CI, 1.008–6.492; *p* = 0.048), ypN (*p* = 0.004), and PDTO drug test (HR, 12.602; 95% CI, 4.024–39.470; *p* < 0.001) were independent risk factors for DFS. Additionally, AJCC TRG (HR, 5.753; 95% CI, 1.737–19.053; *p* = 0.004), ypN (*p* = 0.020), and PDTO drug test (HR, 11.492; 95% CI, 3.274–40.335; *p* < 0.001) were independent risk factors for OS. The multivariate logistic regression analysis results for DFS and OS are presented in Table 3.

**TABLE 3:** Multivariate analyses of clinicopathological characteristics and the organoid drug test with DFS and OS.

Variable	DFS		OS	
	HR (95% CI)	*p* value	HR (95% CI)	*p* value
AJCC TRG^a^ (2 vs. 3)	2.558 (1.008–6.492)	**0.048**	5.753 (1.737–19.053)	**0.004**
Pathological N stage		**0.004**		**0.020**
ypN0	Reference 2		Reference 1	
ypN1	601 (0.812–8.334)	0.108	502 (0.376–6.010)	0.565
ypN2	4.601 (1.587–13.335)	0.005	3.822 (1.099–13.288)	0.035
PDTO drug test	12.602 (4.024–39.470)	**<0.001**	11.492 (3.274–40.335)	**<0.001**

*Note*: Bold values show that AJCC TRG, pathological N stage and PDTO drug test are significant variables.

Abbreviations: AJCC, American Joint Committee on Cancer; CI, confidence interval; DFS, disease‐free survival; HR, hazard ratio; OS, overall survival; PDTO, patient‐derived tumor organoid; TRG, tumor regression grading.

^a^ According to the AJCC guidelines.

**FIGURE 5:**
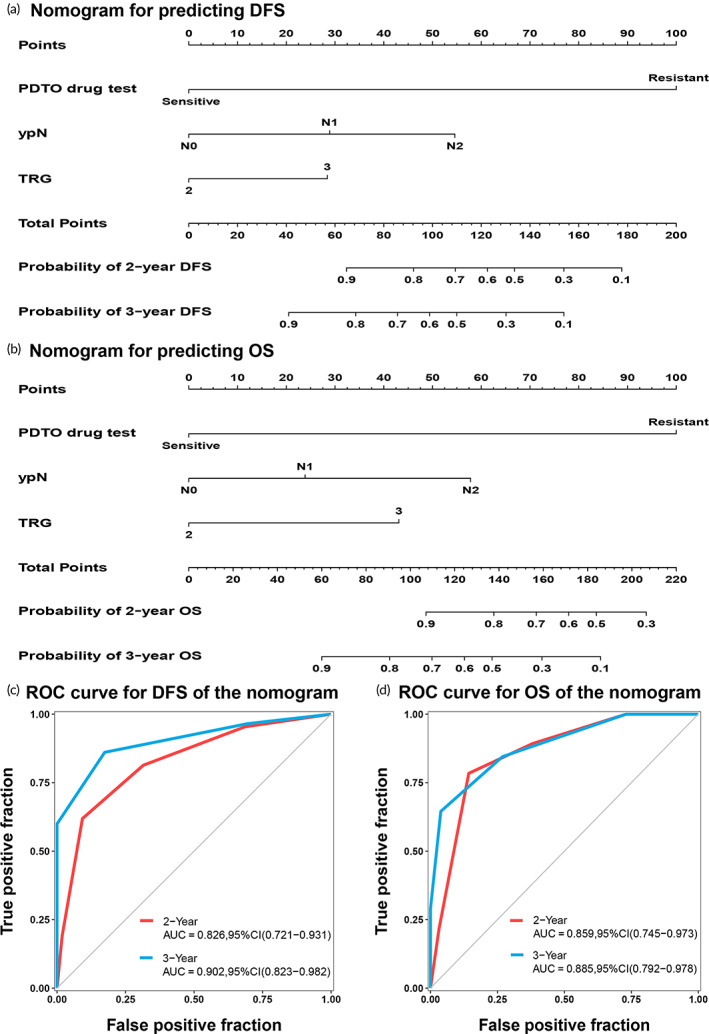
Nomogram with the PDTO drug test and its evaluation. (a) Nomogram with the PDTO drug test for predicting DFS. (b) Nomogram with the PDTO drug test for predicting OS. (c) Time‐dependent ROC curves for 2‐year and 3‐year DFS of the nomogram. (d) Time‐dependent ROC curves for the 2‐year and 3‐year OS of the nomogram. AUC, area under the receiver operating characteristic curve; CI, confidence interval; DFS, disease‐free survival; OS, overall survival; PDTO, patient‐derived tumor organoid; ROC, receiver operating characteristic; TRG, tumor regression grade; ypN, pathological N stage.

Next, we constructed two nomograms incorporating these independent risk factors, including the PDTO drug test to predict DFS (Figure 5a) and OS (Figure 5b). The area under the receiver operating characteristic (ROC) curve (AUC) values for the 2‐year and 3‐year DFS of the nomogram with the PDTO drug test were 0.826 (95% CI, 0.721–0.931) and 0.902 (95% CI, 0.823–0.982) (Figure 5c), respectively. Additionally, nomograms with the PDTO drug test showed AUC values of 0.859 (95% CI, 0.745–0.973) for a 2‐year OS and 0.885 (95% CI, 0.792–0.978) for a 3‐year OS (Figure 5d). Furthermore, the concordance indexes (C‐indexes) of the nomograms for DFS and OS were 0.842 (95% CI, 0.757–0.926) and 0.868 (95% CI: 0.804–0.933), respectively. The calibration curve exhibited excellent concordance between anticipated and observed survival probabilities using the nomogram with the PDTO drug test (Figure S7A, B). Significant improvements in DFS and OS prediction were detected after comparing the clinicopathological model (TRG plus ypN) in the nomogram with the PDTO drug test. The AUC values for the 3‐year DFS between the nomogram with the PDTO drug test and clinicopathological model were 0.902 (95% CI, 0.823–0.982) vs. 0.799 (95% CI, 0.686–0.914), (*p* = 0.002) (Figure S7C), and those for the 3‐year OS were 0.885 (95% CI, 0.792–0.978) versus 0.811 (0.678–0.945) (*p* = 0.043) (Figure S7D). Lastly, the decision curve analysis indicated that the nomograms demonstrated greater net benefits than the clinicopathological models across a majority of reasonable threshold probabilities (Figure S7E,F).

## 3 DISCUSSION

Effective preclinical models for the precise prediction of AC response and prognosis in poor responders after nCRT and surgery for LARC are lacking. Therefore, we used PDTOs as avatars of the patient's tumor to apply chemotherapy drug tests in vitro. Patients were divided into the chemotherapy‐sensitive and chemotherapy‐resistant groups by defining the best cut‐off value of IC50, with significant variances between DFS and OS. Furthermore, the PDTO drug test and valuable clinicopathological risk factors were incorporated into a nomogram, resulting in robust DFS and OS predictions. These results indicate that the PDTO drug test could predict AC benefits for poor responders with LARC after nCRT and surgery.

Developing personalized cancer treatments based on PDTOs has great benefits. Previous studies have reported the successful construction of various PDTOs for preclinical cancer modeling of breast,^19^ liver,^20^ gastric,^21^ nasopharynx,^22^ and colorectal cancers.^23^ However, the materials used to construct these organoids were derived from untreated biopsies or surgical specimens. To our knowledge, this is the first reported platform using irradiated rectal cancer specimens to construct organoids. Our success rate for organoid construction was 62.3%, lower than that of previous studies (75%–90%).^15^, ^17^, ^24^ This disparity may be attributed to the unique characteristics of the irradiated specimens used in our study. In irradiated specimens of rectal cancer, tumor cells may exhibit irregular or scattered distribution under the microscope. Additionally, irradiated rectal cancer specimens often show fibrotic proliferation, and the tumor may be surrounded by proliferative fibrous tissue.^25^ These factors can increase the difficulty of extracting tumor cells and may contribute to a lower success rate of organoid establishment compared to previous studies. We propose two factors related to the success rate of organoid construction using irradiated specimens. First, magnetic resonance imaging (MRI), endoscopy, and other examinations should be carefully reviewed before obtaining specimens to obtain tumor tissue accurately. Second, sufficient time should be allowed for tissue mincing and digestion due to the proliferation of fibrous tissue in irradiated specimens. The digestion process should be closely supervised and moved to the next step when the fragmented tissues can be aspirated through a 1‐mL pipette tip. Overall, we successfully established a platform for constructing PDTOs using irradiated rectal cancer specimens, filling a gap in organoid research. Moreover, our results demonstrate the feasibility of conducting chemotherapy drug sensitivity testing using this platform.

The benefits of AC after nCRT and surgery in LARC are debatable, given the inconsistent results from various trials and studies,^26^, ^27^, ^28^ leading to different recommendations across different guidelines. The European Society for Medical Oncology recommends AC for patients with stage III and high‐risk stage II cancer,^29^ and the National Comprehensive Cancer Network (NCCN) guidelines recommend postoperative chemotherapy for all patients who undergo preoperative chemoradiation.^30^ However, the guidelines in the Netherlands do not endorse the use of AC in patients with rectal cancer.^4^ Nevertheless, it is well established that non‐responders to nCRT have poorer long‐term outcomes, necessitating better patient stratification.^31^ Previous studies have screened clinical and pathological risk factors, showing that the TRG^32^ and TNM stage system^33^ could predict AC benefits; however, their predictive values are limited. Variations in survival outcomes, even in patients with the same TRG and TNM stage receiving similar AC regimens, indicate that novel individualized biomarkers that can identify patients who will benefit from AC are required. Our results revealed that the PDTO drug test is an effective biomarker with an accuracy of 84.88% for predicting the chemotherapy response. Patients with organoid sensitivity were more likely to benefit from AC, and those with organoid resistance experienced limited benefits. Moreover, compared with the traditional clinicopathological factor prediction model, our nomogram based on the PDTO drug test significantly improved the prediction of patients who would benefit from AC. The improvement in the AUC was 11.4% (*p* = 0.002) for the 3‐year DFS and 8.4% (*p* = 0.043) for the 3‐year OS (Figure 4g,h). These data indicate that the nomogram that combines the organoid drug test and clinicopathological factors is useful for identifying patients who will benefit from AC.

Growing evidence supports the use of organoids as ideal preclinical models for patient drug testing. Organoids offer significant advantages over previous models, such as cell lines or PDTX models. Organoids can self‐assemble into three‐dimensional tissue structures that closely resemble the original tissue, ensuring genetic stability, whereas cell lines grow in a two‐dimensional plane, hindering the maintenance of stable genotypes that accurately represent the original tissue and resulting in reduced drug test accuracy.^34^ While PDTX models can form tissue structures, their establishment and drug sensitivity tests typically require several months, posing challenges in guiding timely treatment decisions for patients.^35^ Furthermore, PDTX models exhibit complex cellular compositions, including diverse extracellular matrix components, which can influence drug diffusion and subsequently impact the effectiveness of drug sensitivity testing. In contrast, organoid construction and drug sensitivity testing can yield results within a two‐week timeframe, enabling prompt guidance for subsequent AC. Organoids are not affected by cell type heterogeneity, making them favorable for clinical translation. To exemplify the practical application of organoids, we present a potential scenario: After neoadjuvant treatment, a patient with LARC undergoes surgical resection, and tumor tissues are extracted from the surgical specimens for organoid cultivation and subsequent drug tests. Approximately 15–18 days later, clinicians receive the organoid drug sensitivity results. After about 1 month of patient recovery post‐surgery, clinicians utilize the drug sensitivity results along with clinical pathological findings to tailor personalized adjuvant treatment plans, thereby reducing the risk of recurrence and extending patient survival.

Our research has some drawbacks. First, this was an observational cohort study, which could have introduced selection bias and influenced the accuracy of the statistical analysis. Second, the study was conducted at a single center, limiting its generalizability. Therefore, the PDTO drug test should be further validated in prospective randomized trials that include diverse populations to determine its clinical utility. Third, only the FOLFOX chemotherapy regimen recommended by the NCCN guidelines was tested in this study; therefore, further prospective clinical studies validating the PDTO drug test's ability to determine other chemotherapy regimens a patient may benefit from, such as FOLFOX or FOLFIRI are necessary. Additionally, the success rate of organoid construction in this study was lower compared to that in previous research. In the future, exploring methods for enriching tumor cells in organoids derived from irradiated tumor specimens is warranted. This may involve using high‐resolution imaging techniques to accurately locate tumor cells within the tissue, as well as optimizing tissue digestion and cell extraction procedure.

## 4 MATERIALS AND METHODS

### 4.1 Study design

This cohort study was carried out following the STROBE guidelines. We included patients with a pathologically poor response to nCRT, defined as TRG 2–3 in accordance with the 8th edition of the staging manual developed by the AJCC. Thereafter, data on clinicopathology, prognosis, and PDTO drug tests were extracted from the database, and the optimal IC50 cut‐off value was determined to predict patient response. The efficiency and accuracy of the PDTO IC50 cut‐off value in predicting patient outcomes were determined and the prognostic value of the PDTO drug test in predicting DFS and OS was analyzed in LARC patients following nCRT and surgery.

Institutional Review Board permission was obtained from Nanfang Hospital of Southern Medical University for this investigation. All processes concerning human volunteers were conducted based on the guidelines that are articulated in the Declaration of Helsinki. Each patient gave their informed agreement in writing.

### 4.2 Establishment and identification of organoids from LARC patient samples after chemoradiotherapy

Given the possibility of tumor irregularity following nCRT, preoperative assessment of the presence and location of residual tumors is essential. MRI and endoscopy were conducted preoperatively to achieve precise evaluation. During the surgical procedure, rectal specimens with visible tumors were collected, aided by MRI, endoscopy, and the naked eye (Figure S8). Next, organoid cultures were performed using a previously described protocol with minor modifications.^36^ Briefly, 0.5 × 0.5 × 0.5 cm tumor tissues were retrieved and immediately transported to the laboratory. Then, the tissues were rinsed with Dulbecco's phosphate‐buffered saline containing 5% antibiotics 8–10 times and minced into small fragments of 1–3 mm^3^ using surgical scissors. Next, a mixture of collagenase type II and hyaluronidase was used to digest the tissue fragments in Dulbecco's Modified Eagle Medium/F12 for approximately 1 h at 37°C. After filtration, the suspension was separated by centrifuging it for 3 min at 250 × g. Red blood cells (RBCs) were also extracted using RBC lysis buffer (Invitrogen eBioscience, Waltham, MA, USA). Following collecting, washing, counting, and resuspending the tumor cells in a Matrigel basement membrane matrix (Corning Inc, Corning, NY, USA), 30 μL droplets were dispensed into 48‐well plates and solidified at 37°C for 30 min. After complete gelation, we added colorectal cancer organoid culture medium to each well and kept the cells in an incubator at 37°C with 5% CO_2_. The media was replaced every 2 days, and organoid growth was closely monitored.

### 4.3 Quality control of organoid cultures

Organoids and matched primary tumors were soaked in paraffin followed by H&E staining. Next, immunohistochemical staining was conducted for ki67, cdx‐2, p53, pms2, her‐2, ck20, and ck markers to confirm whether the organoids replicated the original tumor's histological characteristics and marker expression. Moreover, organoid authentication was performed using STR profiling and a Multi‐Amplification Kit (PowerPlex 21 System; Promega, Madison, WI, USA). Notably, organoids may contain mycoplasma contaminants, which resemble organoids when cultured. Therefore, the organoids were routinely checked for mycoplasma contaminants utilizing a MycoAlert Mycoplasma Detection Kit ((#LT07‐218; Lonza, Basel, Switzerland).

### 4.4 Drug sensitivity tests of organoids from LARC samples from patients after chemoradiotherapy

Organoids were split 1:3 to achieve the required cell numbers for drug sensitivity tests. For subsequent drug sensitivity analyses, organoids in good condition were dissociated into single cells using 1x TrypLE (Gibco, Billings, MT, USA), resuspended in 5% Matrigel/organoid culture media (500–1000 cells/μL), and distributed onto 96‐well plates (10 μL/well). Organoids were kept for 3 days to recover before commencing treatment. Next, we tested PDTOs with fluorouracil and oxaliplatin (1:1), emulating the clinical XELOX/FOLFOX chemotherapy regimen. The concentrations used were 200 μmol/L–0.20 μmol/L from a six‐point four‐fold dilution, and each point was tested in technical triplicate. Following 96 h of drug treatment, the CellTiter‐Glo 3D Cell Viability Assay (Promega, Madison, WI, USA) was conducted as reported previously,^16^ and luminescence signals were measured as indicators of cell viability. By comparing the fluorescence values as percentages relative to the vehicle group, the relative changes in organoid response to different drug concentrations can be assessed. Additionally, dose–response curves and IC50 values were determined by fitting nonlinear dose–response regression using GraphPad Prism software (GraphPad, San Diego, CA, USA). Figure S9 shows the protocols for establishing rectal cancer organoids to drug testing.

Furthermore, cell death in the organoids was detected by staining PDTOs with 1 mg/mL Hoechst 33258 (Sigma‐Aldrich Japan K.K. Tokyo, Japan) and 1.5 μmol/L propidium iodide (Sigma‐Aldrich Japan K.K. Tokyo, Japan) 1 h before imaging, as described previously.^37^ Finally, organoids were imaged using an imaging system based on a Carl Zeiss LSM510 laser scanning microscope and analyzed using the Zen analysis software (Zeiss, Oberkochen, Germany). The number of live and dead cells was quantified using Image J software and Image pro‐plus 6.0 (Media Cybernetics, Rockville, MD, USA). Microscopic images depicting the organoids before and after treatment were captured. The size of organoids before and after treatment was measured using Image J software (Media Cybernetics, Rockville, MD, USA).

### 4.5 Study population

We extracted clinical data from the database of patients with LARC who underwent curative resection after nCRT and had tumor organoid culture findings, with drug sensitivity tests between October 2018 and December 2021 at Nanfang Hospital. Inclusion criteria were (1) age 18–75 years, (2) postoperative specimens pathologically confirmed as carcinoma and TRG 2–3; (3) tumor tissues successfully extracted for organoid culture and drug sensitivity tests. Exclusion criteria were (1) American Society of Anesthesiology (ASA) score >3; (2) failure to culture organoids or contamination; (4) distant metastasis; (5) multiple tumors; and (6) refusal or inability to complete the standardized XELOX/FOLFOX AC regimen postoperatively.

Demographic information recorded at baseline included age, sex, body mass index, ASA score, carcinoembryonic antigen (CEA) level, mrIMF, mrEMVI, tumor size, tumor location, AJCC stage before nCRT, clinical T stage (cT), clinical N stage (cN), surgical methods and differentiation, pathological AJCC stage, ypT, ypN, and TRG.

The treatment for all patients was based on the NCCN guideline for rectal cancer. In combination with long‐term radiation at an overall dosage of 50.4 Gy in 28 fractions, nCRT comprised 5‐Fu or capecitabine‐based chemotherapy, and AC was administered with FOLFOX or XELOX. A CEA value ≥5 ng/mL was considered high. Furthermore, the tumor's size was evaluated by calculating its length and thickness on MRI. Additionally, tumor location was evaluated by measuring the length between the anal verge and the distal margin at the moment of the examination. Positive mrIMF refers to the length between the tumor and mesorectal fascia ≤1 mm.^38^ Moreover, in accordance with the 2010 WHO classification of digestive tumors, classification was as follows: well (G 1), moderate (G 2), and low (G 3).^39^ Furthermore, the eighth version of the AJCC staging manual was used to evaluate TRG.^40^ Desmoplastic response associated with the presence of residual cancer was considered TRG 2, while minor tumor response evidence was classified as TRG 3. Postoperatively, patients were observed once every 3 months in the initial 2 years, every 6 months in the following 3 years, and yearly after that. The duration of follow‐up was determined from the surgical procedure date until the most recent follow‐up or date of death. During the final follow‐up, the survival condition was recorded.

### 4.6 Association of the organoid drug response with patient survival

The ideal cut‐off value for IC50 was identified utilizing maximally selected rank statistics based on individual IC50 values for PDTO drug tests and patients' clinical responses. Patients were classified into organoid‐sensitive and organoid‐resistant subgroups based on the threshold value. The effect of the PDTO drug test on DFS and OS was evaluated using Kaplan–Meier survival analysis.

### 4.7 Construction of a predictive nomogram

The univariate Cox regression analysis included the PDTO drug test and all clinicopathological variables. Additionally, in the multivariate Cox regression analysis, only factors with *p*‐values less than 0.05 were chosen to evaluate the possible connections between the organoid drug response and clinicopathological characteristics with DFS and OS. Subsequently, a novel predictive model—a nomogram with the PDTO drug test—was developed. Next, discrimination and calibration were conducted to evaluate the reliability of the nomogram as a predictive tool. Additionally, the performance of nomogram discrimination was measured using the concordance index (C‐index) in addition to receiver operating characteristic curve (ROC) analyses. Lastly, calibration curves were plotted using Hosmer–Lemeshow tests to visually depict the calibration of the nomogram and assess the goodness of fit. A significant test result indicated that the model's calibration was imperfect.

### 4.8 Statistical analysis

Categorical findings were analyzed using the chi‐square or Fisher's exact tests, whereas continuous findings were analyzed using the unpaired Student's t‐test or the Mann–Whitney U test. Additionally, Cox proportional hazards regression was utilized to examine DFS and OS univariate and multivariate. SPSS 26.0 (SPSS, Chicago, IL, USA) and R version 4.0.5 (R Foundation for Statistical Computing, Vienna, Austria) were used for the statistical analyses. Furthermore, survival analyses and maximally selected rank statistics were performed using the “survminer” and the “survival” packages. Next, the area under each ROC curve was calculated using the “pROC” package. Lastly, we used the “rms” package to develop, validate, and assess the performance of the prognostic nomogram. Statistical power analysis was made by using PASS 2021 software. A *p* value of <0.05 was determined to be statistically significant.

## 5 CONCLUSIONS

We established a PDTO drug test platform using irradiated rectal cancer specimens and found that the PDTO drug test was significantly associated with the prognosis of patients who underwent nCRT followed by surgery and AC. Moreover, by incorporating the PDTO drug test with clinicopathological risk factors, two nomograms were created and validated, improving the prediction of LARC prognosis compared with the clinicopathological risk factor system alone. Furthermore, the PDTO drug test could identify LARC patients with TRG 2 and 3 after nCRT who would benefit from AC, indicating its immense potential for clinical applications.

## AUTHOR CONTRIBUTIONS

**Weisong Xue:** Conceptualization (equal); data curation (equal); formal analysis (equal); investigation (equal); methodology (equal); software (equal); visualization (equal); writing – original draft (equal). **Ting Wang:** Data curation (equal); investigation (equal); software (equal); writing – original draft (equal). **Jiaxin Yao:** Data curation (equal); investigation (equal); methodology (equal); writing – original draft (equal). **Wei Wu:** Formal analysis (equal); investigation (equal); software (equal); writing – original draft (equal). **Dexin Chen:** Data curation (equal); formal analysis (equal); validation (equal). **Botao Yan:** Formal analysis (equal); investigation (equal); visualization (equal). **Xiaoyu Dong:** Data curation (equal); investigation (equal); methodology (equal); validation (equal). **Yuting Tang:** Data curation (equal); formal analysis (equal); investigation (equal); methodology (equal). **Yunli Zeng:** Data curation (equal); investigation (equal); methodology (equal). **Yueyu He:** Formal analysis (equal); investigation (equal); methodology (equal). **Peihua Cao:** Data curation (equal); formal analysis (equal); software (equal); validation (equal). **Fangyuan Shao:** Conceptualization (equal); methodology (equal); supervision (equal); writing – review and editing (equal). **Wenhua Huang:** Conceptualization (equal); funding acquisition (equal); project administration (equal); supervision (equal); writing – review and editing (equal). **Chuxia Deng:** Conceptualization (equal); supervision (equal); validation (equal); writing – review and editing (equal). **Jun Yan:** Conceptualization (equal); funding acquisition (equal); methodology (equal); resources (equal); supervision (equal); writing – review and editing (equal).

## FUNDING INFORMATION

The authors would like to thank the pathologist Guanglong Liu for his valuable help in the operation and interpretation of H&E and immunohistochemistry. This work was supported by grants from the National Natural Science Foundation of China [grant numbers 82273360, 31972915, and 32271181], the National Key R&D Program of China [grant number 2022YFB4600600], the Guangdong Provincial Major Talents Project [grant number 2019JC05Y361], the Fu Jian Medical Innovation Project [grant number 2022CXA028], the Science and Technology Planning Project of Guangzhou City [grant number 202206010085], the China Postdoctoral Science Foundation [grant number 2020 M682789], the Clinical Research Project of Nanfang Hospital [grant numbers 2018CR034, 2020CR001, and 2020CR011], the President Foundation of Nanfang Hospital, Southern Medical University [grant number 2019Z023], the Training Program for Undergraduate Innovation and Entrepreneurship [grant numbers 202212121011, S202212121092, and S202212121104], and the Guangdong Basic and Applied Basic Research Foundation [grant number 2020B1515120001].

## CONFLICT OF INTEREST STATEMENT

The authors have no interests to declare.

### PEER REVIEW

The peer review history for this article is available at https://www.webofscience.com/api/gateway/wos/peer‐review/10.1002/btm2.10586.

## Supporting information


**Data S1.** Supporting Information.

**Figure S1.** Mycoplasma detection assay results. (A) The kinetics of ATP generation in mycoplasma‐negative PDTO cultures. (B) Box plot of the MycoAlert ratios of all tested PDTOs. PDTO, patient‐derived tumor organoids.
**Figure S2.** STR test results of PDTO and paired tumor tissue from one patient. A‐B. STR profiles of PDTOs and paired tumor tissues. A single peak is displayed when two alleles of homologous chromosomes at the same gene locus are identical, and two peaks are displayed when two alleles of homologous chromosomes at the same gene locus are different. C. Comparison of STR profiles between PDTOs and paired tumor tissues. Evaluation value (EV) = (number of generated peaks of cell line from PDTOs) × 2/(total number peaks of cell lines from PDTOs and paired tumor tissues). PDTO, patient‐derived tumor organoids; STR, short tandem repeat.
**Figure S3.** Immunohistochemistry staining of p53, pms2, and ck20 on PDTOs and corresponding primary tumors (×200 magnification, 100 μm scale bars). PDTO, patient‐derived tumor organoids.
**Figure S4.** Quantification of LIVE/DEAD cell staining. The number of live cells in the drug‐sensitive organoids was significantly lower than that in the drug‐resistant group, whereas the number of dead cells in the drug‐sensitive organoids was significantly higher than that in the drug‐resistant group. ※*p*<0.05.
**Figure S5. (**A) Optical images of organoids before and after treatment with different drug concentrations (×40 magnification). (B) The size of the organoids before and after treatment with different concentrations of drug in both sensitive group and resistant group. The size of organoids sensitive to chemotherapy drugs was significantly decreased as the drug concentration increased, whereas the size of drug‐resistant organoids was decreased in a slower manner with increasing drug concentration.
**Figure S6.** The dose–response curve of cell viability in patient‐derived organoids in both the sensitive and resistant groups in response to six different concentrations of FOLFOX chemotherapy.
**Figure S7.** (A) Calibration curves for 2‐year and 3‐year DFS of the nomogram. (B) Calibration curves for 2‐year and 3‐year OS of the nomogram. (C) Comparison of AUCs for 3‐year DFS between the nomogram with the PDTO drug test and the clinicopathological model. (D) Comparison of AUCs for 3‐year OS between the nomogram with the PDTO drug test and the clinicopathological model. (E) Decision curve analysis of DFS for different models. (F) Decision curve analysis of OS for different models. AUC, area under the receiver operating characteristic curve; DFS, disease‐free survival; OS, overall survival; PDTO, patient‐derived tumor organoid.
**Figure S8.** Enteroscopy, MRI, and surgical resection of rectal specimens from one patient. The residual tumor can be observed on MRI, enteroscopy, and the specimen. (red arrow) Postoperative pathology confirmed the tumor to be TRG 3. Tumor tissue was retrieved in this patient, and an organoid was successfully constructed. MRI, magnetic resonance imaging; TRG, tumor regression grade.
**Figure S9.** PDTO drug test protocol. Different protocol steps, from establishing rectal cancer organoids to drug testing. PDTO, patient‐derived tumor organoids.

**Table S1.** Univariate analyses of clinicopathological characteristics and the organoid drug test with DFS and OS.

## Data Availability

The data in the current study are available from the corresponding author upon reasonable request.
