# Supplementary material for: Use of patient‐derived tumor organoid platform to predict the benefit of postoperative adjuvant chemotherapy for poor responders to neoadjuvant chemoradiotherapy in locally advanced rectal cancer
Source: Bioeng Transl Med. 2023 Aug 16;8(6):e10586. doi: 10.1002/btm2.10586 (PMC10658544; doi:10.1002/btm2.10586)
Supplement: Supplementary file 1 — Data S1. Supporting Information. [file BTM2-8-e10586-s002.docx]

Power analysis:

1. For comparing the response rates between organoid-sensitive and organoid-resistant groups, the sample sizes of 54 in sensitive group and 32 in resistant group achieve 100% power to detect a difference between the group response rates of 0.645 using a two-sided Z-test at a significance level of 0.05.
2. For survival analysis of DFS, a two-sided log-rank test with an overall sample size of 86 subjects (54 in sensitive group and 32 in resistant group) achieves 100% power at a 0.05 significance level to detect a hazard ratio of 17.564. Similarly, the sample size also achieves 100% in terms of OS analysis.
3. We also calculated the powers when constructing different nomograms in predicting DFS and OS (Table R1). For example, our nomogram model achieves 99.92% power from 27 events out of 86 samples in 2-year DFS to detect a difference of 0.402 between an AUROC under the null hypothesis of 0.5 and an AUROC under the alternative hypothesis of 0.902 using a two-sided test at a significance level of 0.05.

Thus, the sample size can achieve enough power in our study.

Table R1. Statistical power for nomograms in predicting DFS and OS.

|  | AUROC | Observed Events | Power (%) |
| --- | --- | --- | --- |
| 2-year DFS | 0.826 | 21 | 99.92 |
| 3-year DFS | 0.902 | 27 | 100.00 |
| 2-year OS | 0.859 | 10 | 98.67 |
| 3-year OS | 0.885 | 21 | 100.00 |
