# Supplementary material for: Use of patient‐derived tumor organoid platform to predict the benefit of postoperative adjuvant chemotherapy for poor responders to neoadjuvant chemoradiotherapy in locally advanced rectal cancer
Source: Bioeng Transl Med. 2023 Aug 16;8(6):e10586. doi: 10.1002/btm2.10586 (PMC10658544; doi:10.1002/btm2.10586)
Supplement: Supplementary file 2 — Figure S1. Mycoplasma detection assay results. (A) The kinetics of ATP generation in mycoplasma‐negative PDTO cultures. (B) Box plot of the MycoAlert ratios of all tested PDTOs. PDTO, patient‐derived tumor organoids. Figure S2. STR test results of PDTO and paired tumor tissue from one patient. A‐B. STR profiles of PDTOs and paired tumor tissues. A single peak is displayed when two alleles of homologous chromosomes at the same gene locus are identical, and two peaks are displayed when two alleles of homologous chromosomes at the same gene locus are different. C. Comparison of STR profiles between PDTOs and paired tumor tissues. Evaluation value (EV) = (number of generated peaks of cell line from PDTOs) × 2/(total number peaks of cell lines from PDTOs and paired tumor tissues). PDTO, patient‐derived tumor organoids; STR, short tandem repeat. Figure S3. Immunohistochemistry staining of p53, pms2, and ck20 on PDTOs and corresponding primary tumors (×200 magnification, 100 μm scale bars). PDTO, patient‐derived tumor organoids. Figure S4. Quantification of LIVE/DEAD cell staining. The number of live cells in the drug‐sensitive organoids was significantly lower than that in the drug‐resistant group, whereas the number of dead cells in the drug‐sensitive organoids was significantly higher than that in the drug‐resistant group. ※p<0.05. Figure S5. (A) Optical images of organoids before and after treatment with different drug concentrations (×40 magnification). (B) The size of the organoids before and after treatment with different concentrations of drug in both sensitive group and resistant group. The size of organoids sensitive to chemotherapy drugs was significantly decreased as the drug concentration increased, whereas the size of drug‐resistant organoids was decreased in a slower manner with increasing drug concentration. Figure S6. The dose–response curve of cell viability in patient‐derived organoids in both the sensitive and resistant groups in response to six differe [file BTM2-8-e10586-s001.docx]

**Supplementary Figures**


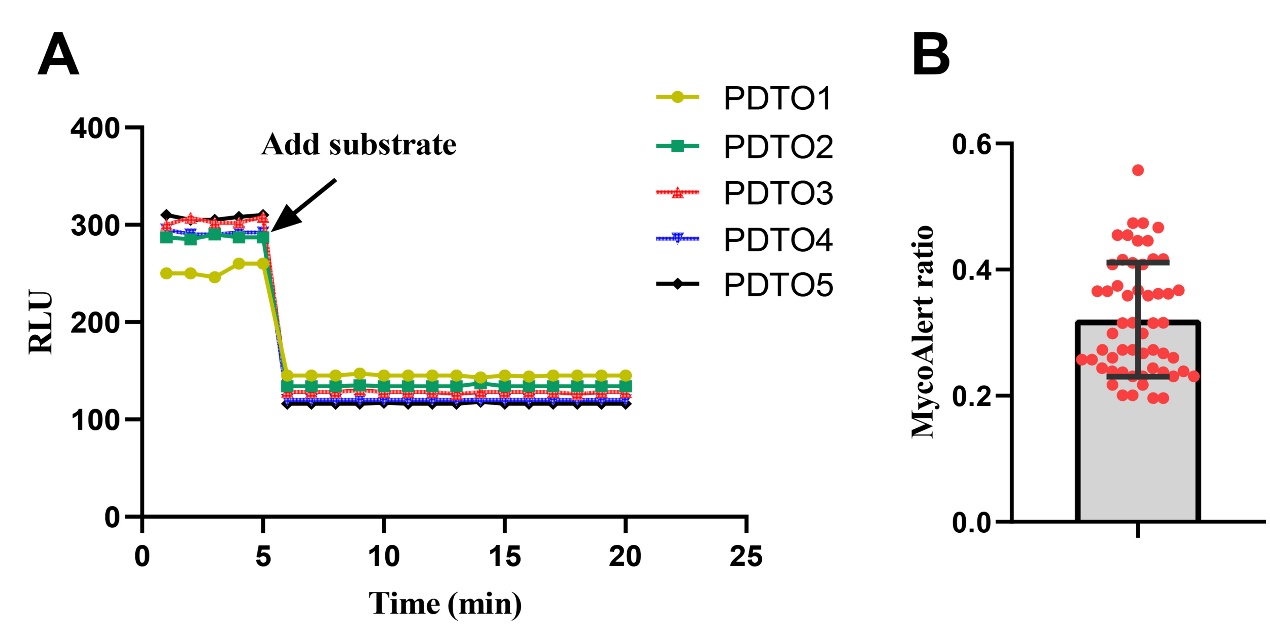


**Supplementary Figure 1.** Mycoplasma detection assay results. (A) The kinetics of ATP generation in mycoplasma-negative PDTO cultures. (B) Box plot of the MycoAlert ratios of all tested PDTOs. PDTO, patient-derived tumor organoids.


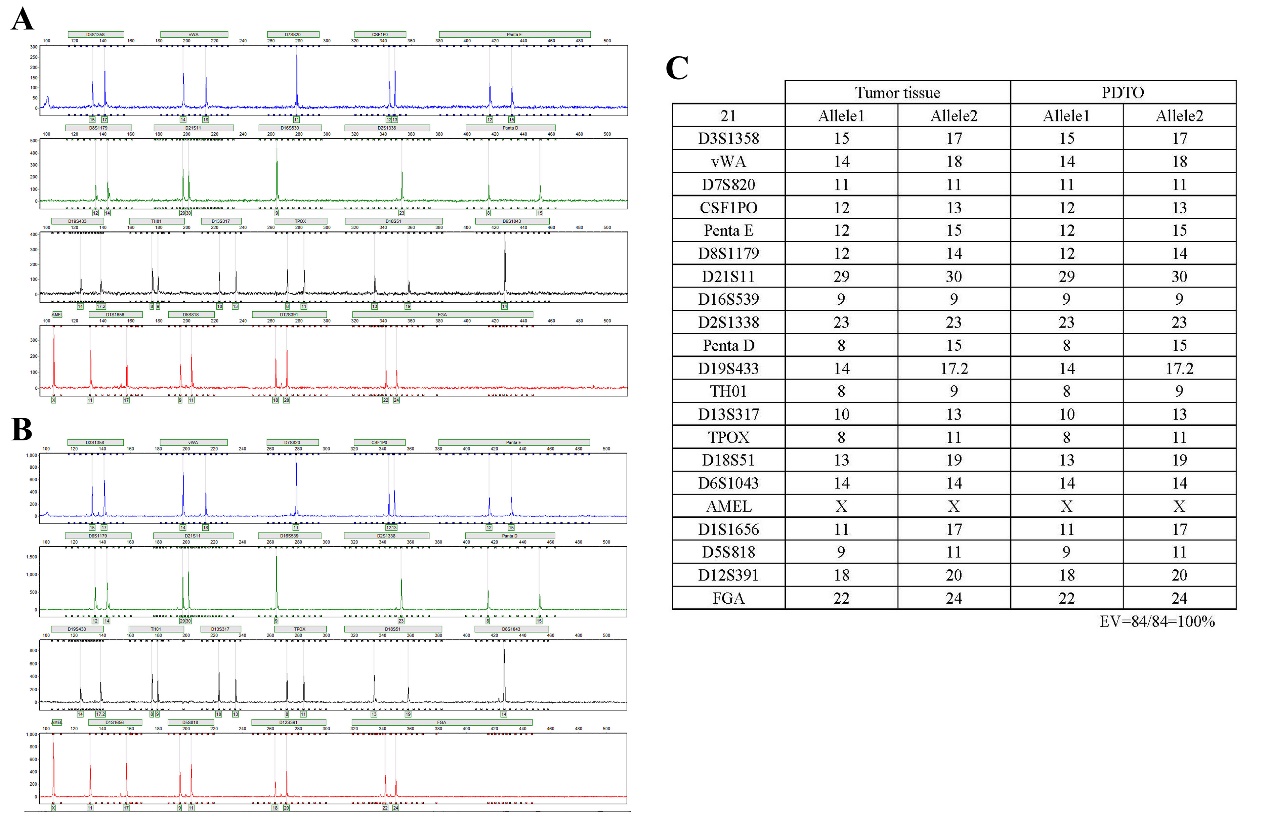


**Supplementary Figure. 2.** STR test results of PDTO and paired tumor tissue from one patient. A-B. STR profiles of PDTOs and paired tumor tissues. A single peak is displayed when two alleles of homologous chromosomes at the same gene locus are identical, and two peaks are displayed when two alleles of homologous chromosomes at the same gene locus are different. C. Comparison of STR profiles between PDTOs and paired tumor tissues. Evaluation value (EV) = (number of generated peaks of cell line from PDTOs)×2/(total number peaks of cell lines from PDTOs and paired tumor tissues). PDTO, patient-derived tumor organoids; STR, short tandem repeat.


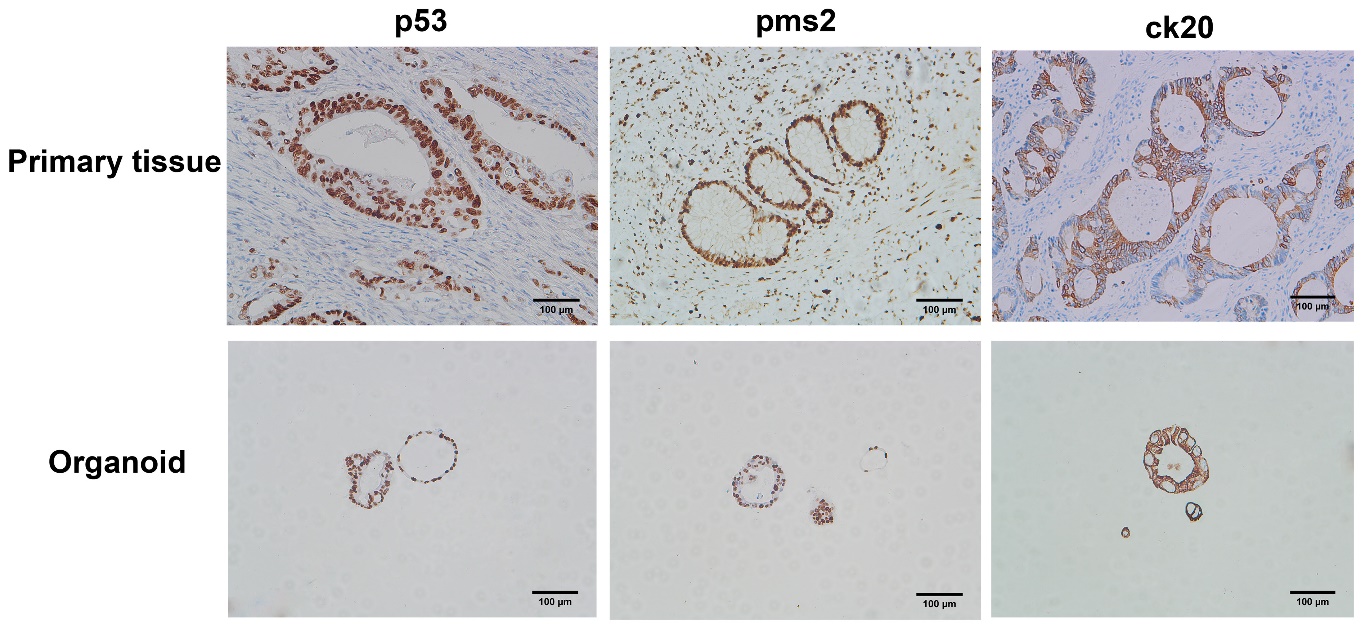


**Supplementary Figure 3.** Immunohistochemistry staining of p53, pms2, and ck20 on PDTOs and corresponding primary tumors (×200 magnification, 100 μm scale bars). PDTO, patient-derived tumor organoids.

**
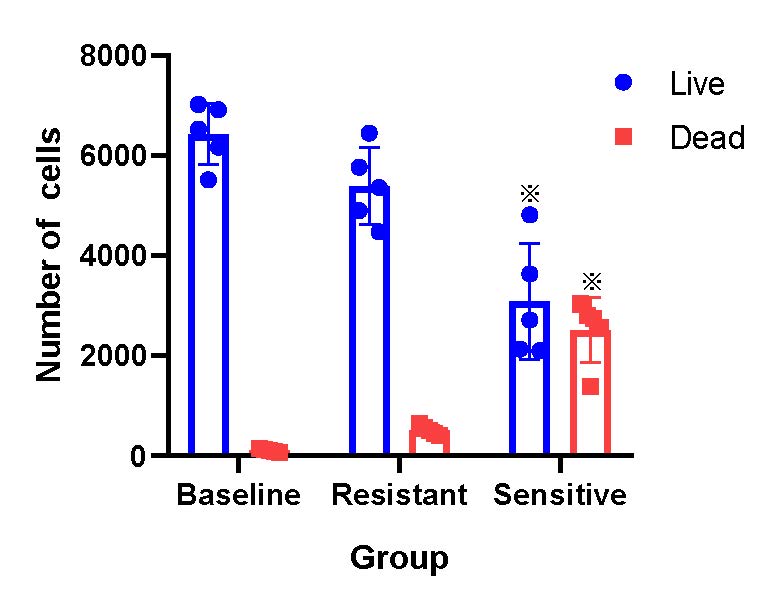
**

**Supplementary Figure 4.** Quantification of LIVE/DEAD cell staining. The number of live cells in the drug-sensitive organoids was significantly lower than that in the drug-resistant group, whereas the number of dead cells in the drug-sensitive organoids was significantly higher than that in the drug-resistant group. ※ *P*＜0.05.

**
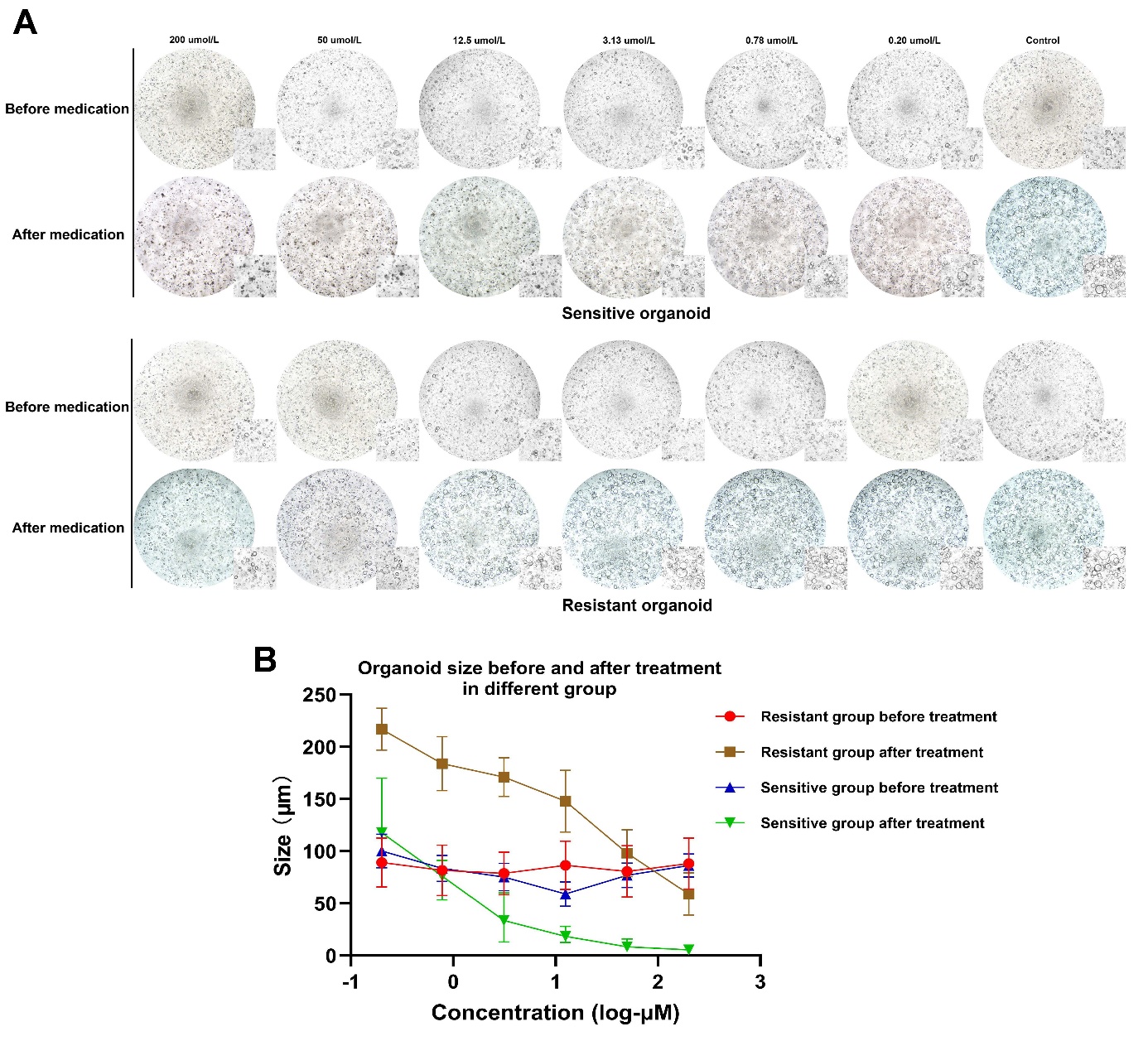
**

**Supplementary Figure 5.** A. Optical images of organoids before and after treatment with different drug concentrations (×40 magnification). B. The size of the organoids before and after treatment with different concentrations of drug in both sensitive group and resistant group. The size of organoids sensitive to chemotherapy drugs was significantly decreased as the drug concentration increased, whereas the size of drug-resistant organoids was decreased in a slower manner with increasing drug concentration.


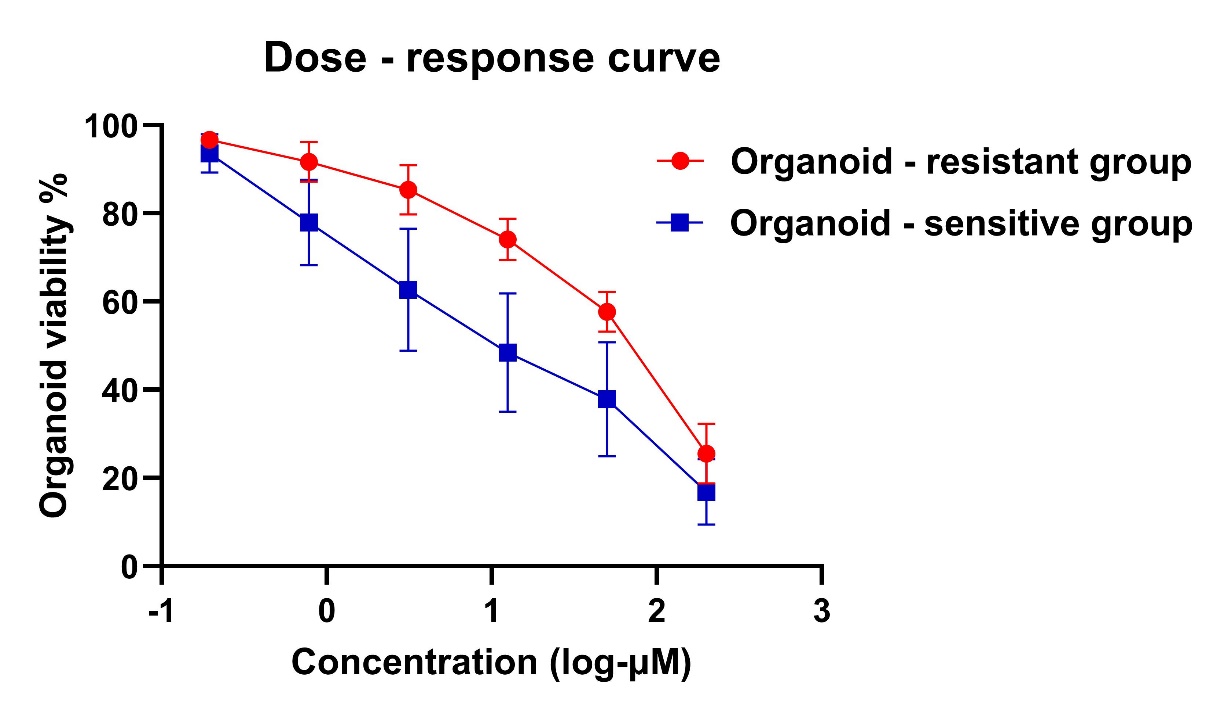


**Supplementary Figure 6.** The dose-response curve of cell viability in patient-derived organoids in both the sensitive and resistant groups in response to six different concentrations of FOLFOX chemotherapy.


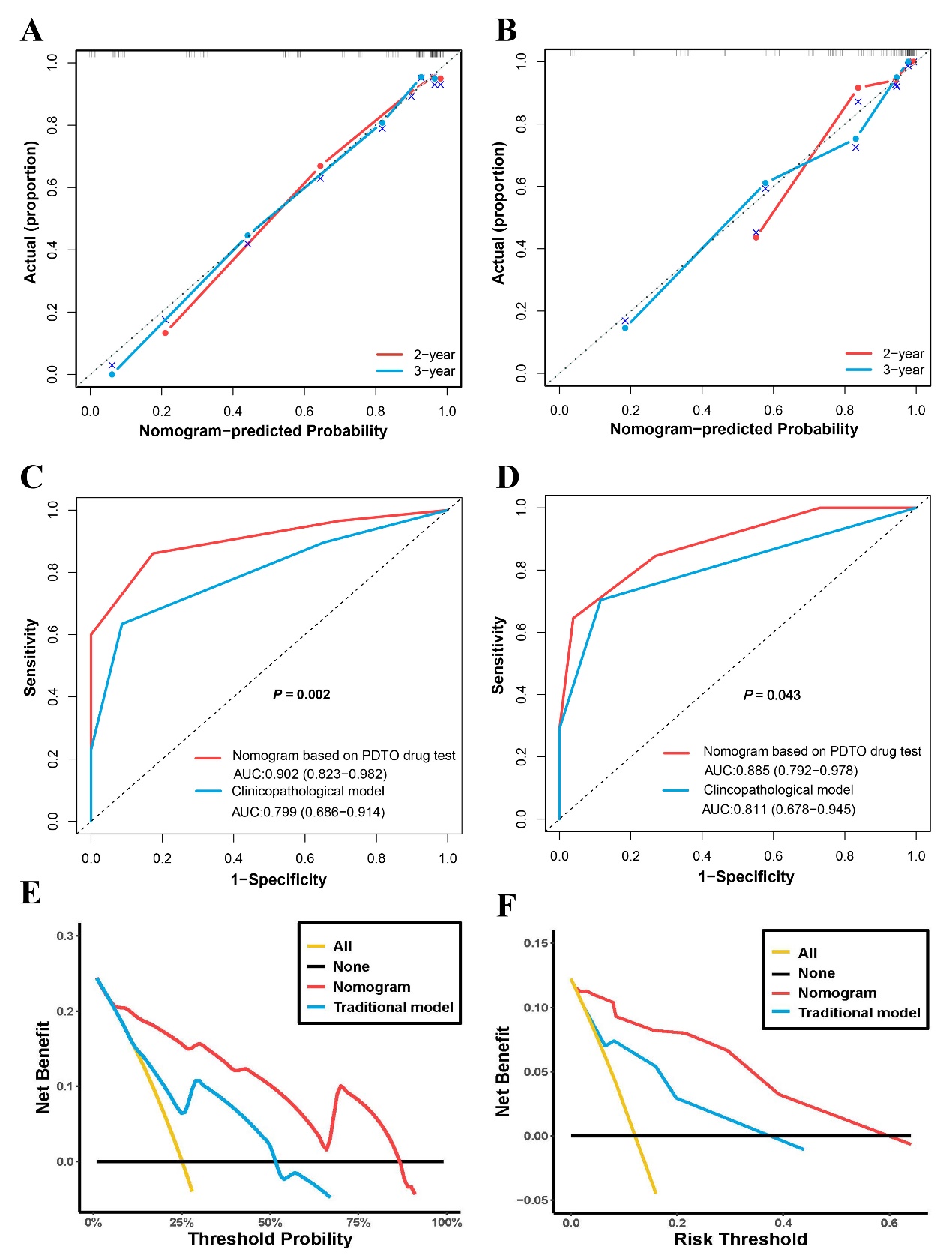


**Supplementary Figure 7.** (A) Calibration curves for 2-year and 3-year DFS of the nomogram. (B) Calibration curves for 2-year and 3-year OS of the nomogram. (C) Comparison of AUCs for 3-year DFS between the nomogram with the PDTO drug test and the clinicopathological model. (D) Comparison of AUCs for 3-year OS between the nomogram with the PDTO drug test and the clinicopathological model. (E) Decision curve analysis of DFS for different models. (F) Decision curve analysis of OS for different models. AUC, area under the receiver operating characteristic curve; DFS, disease-free survival; OS, overall survival; PDTO, patient-derived tumor organoid.


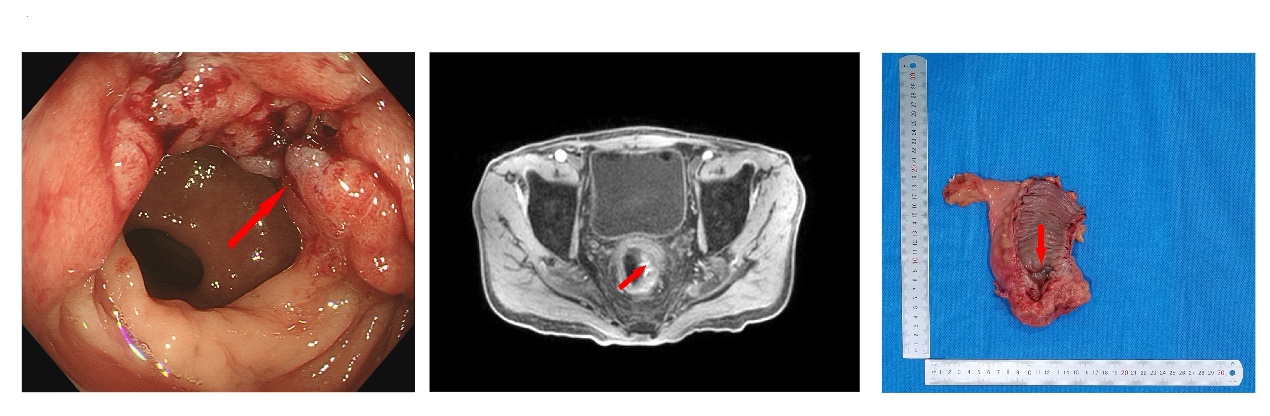


**Supplementary Figure 8.** Enteroscopy, MRI, and surgical resection of rectal specimens from one patient. The residual tumor can be observed on MRI, enteroscopy, and the specimen. (red arrow) Postoperative pathology confirmed the tumor to be TRG 3. Tumor tissue was retrieved in this patient, and an organoid was successfully constructed. MRI, magnetic resonance imaging; TRG, tumor regression grade.


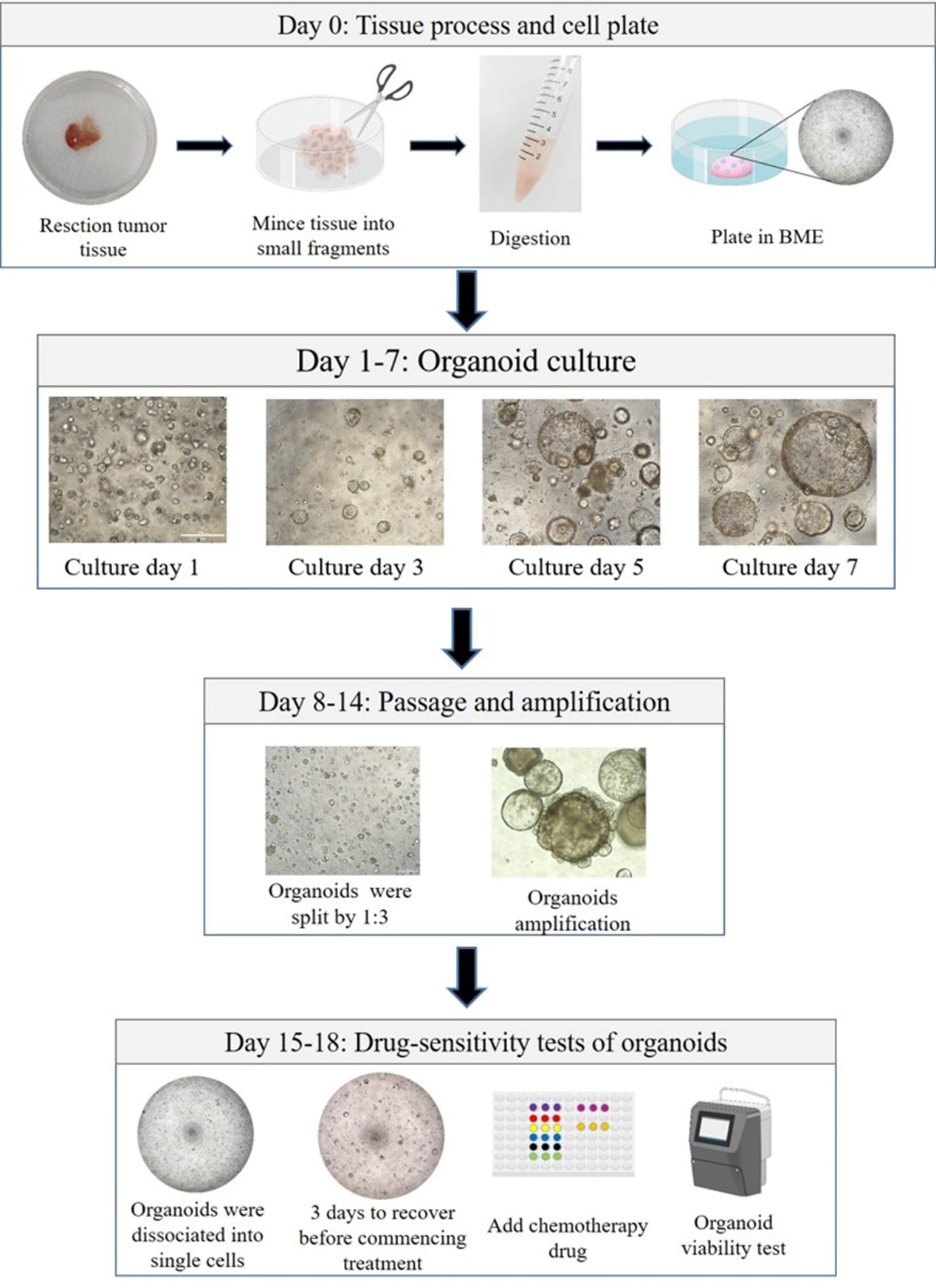


**Supplementary Figure 9. PDTO drug test protocol.** Different protocol steps, from establishing rectal cancer organoids to drug testing. PDTO, patient-derived tumor organoids.
