# Supplementary material for: Use of patient‐derived tumor organoid platform to predict the benefit of postoperative adjuvant chemotherapy for poor responders to neoadjuvant chemoradiotherapy in locally advanced rectal cancer
Source: Bioeng Transl Med. 2023 Aug 16;8(6):e10586. doi: 10.1002/btm2.10586 (PMC10658544; doi:10.1002/btm2.10586)
Supplement: Supplementary file 3 — Table S1. Univariate analyses of clinicopathological characteristics and the organoid drug test with DFS and OS. [file BTM2-8-e10586-s003.docx]

| **Supplementary Table 1.** Univariate analyses of clinicopathological characteristics and the organoid drug test with DFS and OS | | | | |
| --- | --- | --- | --- | --- |
| **Variable** | **DFS** | | **OS** | |
|  | **HR (95% CI)** | ***P* value** | **HR (95% CI)** | ***P* value** |
| **Sex** (female vs. male) | 1.364 (0.611–3.045) | 0.448 | 0.932 (0.355–2.443) | 0.887 |
| **Age** (years) | 0.977 (0.939–1.016) | 0.243 | 0.986 (0.942–1.032) | 0.550 |
| **BMI** (kg/m^2^) | 0.949 (0.838–1.075) | 0.411 | 0.928 (0.809–1.065) | 0.289 |
| **ASA score** (I vs. II) | 1.144 (0.500–2.614) | 0.750 | 0.816 (0.338–1.971) | 0.652 |
| **CEA** (≥5 ng/mL vs. <5 ng/mL) | 1.417 (0.571–3.515) | 0.452 | 0.731 (0.214–2.497) | 0.617 |
| **Tumor location^※^** (mm) | 1.541 (0.720–3.297) | 0.265 | 1.462 (0.616–3.473) | 0.389 |
| **Tumor length** (mm) | 1.016 (0.994–1.038) | 0.149 | 1.013 (0.987–1.040) | 0.326 |
| **Tumor thickness** (mm) | 1.033 (0.998–1.071) | 0.067 | 1.020 (0.982–1.061) | 0.309 |
| **mrEMVI** (yes vs. no) | 3.380 (1.575–7.255) | **0.002** | 3.657 (1.550–8.631) | **0.003** |
| **mrIMF** (positive vs. negative) | 2.965 (1.346–6.535) | **0.007** | 3.690 (1.548–8.798) | **0.003** |
| **AJCC stage* before nCRT** (II vs. III) | 1.832 (0.432–7.759) | 0.411 | 1.528 (0.355–6.576) | 0.570 |
| **Clinical T stage**  cT3  cT4a  cT4b | Reference  1.264 (0.562–2.847)  2.643 (0.726–9.621) | 0.216  0.571  0.140 | Reference  1.547 (0.609–3.932)  2.621 (0.382–12.705) | 0.201  0.359  0.232 |
| **Clinical N stage**  cN0  cN1  cN2  cNx | Reference  1.073 (0.125–9.213)  1.588 (0.211–11.926)  0.699 (0.063–7.724) | 0.999  0.949  0.653  0.770 | Reference  0.900 (0.125–6.459)  1.200 (0.182–7.926)  1.776 (0.343–9.200) | 0.868  0.917  0.850  0.494 |
| **Surgery** (SPS vs. APR) | 0.796 (0.239–2.648) | 0.710 | 0.937 (0.275–3.191) | 0.917 |
| **Differentiation** (well+moderate vs. low)  **Circumferential margin** (positive vs. negative)  **Vascular invasion** (yes vs. no)  **Nerve invasion** (yes vs. no) | 0.897 (0.310–2.593)  0.797 (0.108–5.878)  1.651 (0.696–3.915)  1.143 (0.522–2.500) | 0.840  0.824  0.255  0.737 | 0.717 (0.211–2.445)  1.252 (0.167–9.386)  1.954 (0.745–5.128)  1.161 (0.481–2.803) | 0.596  0.827  0.173  0.740 |
| **AJCC stage*** |  | **<0.001** |  | **0.023** |
| I | Reference |  | Reference |  |
| II | 1.500 (1.154–7.566) | 0.019 | 1.677 (1.061–3.521) | 0.036 |
| III | 6.263 (2.697–16.248) | <0.001 | 4.875 (1.481–6.053) | <0.001 |
| **AJCC TRG*** (2 vs. 3) | 2.619 (1.198–5.726) | **0.016** | 3.470 (1.345–8.951) | **0.010** |
| **Pathological T stage**  ypT2  ypT3  ypT4 | Reference  1.763 (0.492–6.323)  3.686 (1.048–12.968) | **0.046**  0.384  0.042 | Reference  1.534 (0.413–5.695)  2.374 (0.640–8.804) | 0.165  0.523  0.196 |
| **Pathological N stage**  ypN0  ypN1  ypN2 | Reference  2.357 (0.897–6.193)  7.121 (2.62–19.047) | **<0.001**  0.082  <0.001 | Reference  2.024 (0.641–6.391)  7.003 (2.313–21.203) | **<0.001**  0.229  <0.001 |
| **PDTO drug test** | 17.563 (5.988–51.518) | **<0.001** | 15.646 (4.592–53.311) | **<0.001** |
| *DFS* disease-free survival, *OS* overall survival, *HR* hazard ratio, *CI* confidence interval*,* *BMI* body mass index, *ASA* American Society of Anesthesiologists, *CEA* carcinoembryonic antigen, *mrEMVI* MRI-detected extramural vascular invasion, *mrIMF* MRI-detected involved mesorectal fascia, *AJCC* American Joint Committee on Cancer*,* *nCRT* neoadjuvant chemoradiotherapy, *SPS* sphincter preservation surgery, *APR* abdominoperineal resection, *TRG* tumor regression grading, *PDTO* patient-derived tumor organoid. ^※^ Distance of the tumor from the anal verge. * According to the AJCC guidelines. | | | | |
